# Bio-compatible organic humidity sensor transferred to arbitrary surfaces fabricated using single-cell-thick onion membrane as both the substrate and sensing layer

**DOI:** 10.1038/srep30065

**Published:** 2016-07-20

**Authors:** Memoon Sajid, Shahid Aziz, Go Bum Kim, Soo Wan Kim, Jeongdai Jo, Kyung Hyun Choi

**Affiliations:** 1 Department of Mechatronics Engineering, Jeju National University, Jeju 690-756, South Korea; 2 Korean Institute of Machinery and Materials, Yuseong-Gu, Daejeon 305-343, Republic of Korea

## Abstract

A bio-compatible disposable organic humidity sensor has been fabricated that can be transferred to any arbitrary target surface. Single cell thick onion membrane has been used as the substrate while it also doubles as the active layer of the sensor. Two different types of sensors were fabricated. In type-1, the membrane was fixed into a plastic frame with IDT patterns on one side while the other side was also exposed to environment. In type-2, onion membrane was attached to a glass substrate with one side exposed to environment having an IDT screen-printed on top of it. The electrical output response of the sensors showed their ability to detect relative humidity between 0% RH and 80% RH with stable response and good sensitivity. The impedance of the sensors changed from 16 MΩ to 2 MΩ for type-1 and 6 MΩ to 20 KΩ for type-2. The response times of type-1 and type-2 were ~1 and 1.5 seconds respectively. The recovery times were ~10.75 seconds and ~11.25 seconds for type-1 and type-2 respectively. The device was successfully transferred to various randomly shaped surfaces without damaging the device.

Environmental sensing has been widely used to detect various quantities like temperature, humidity, different gases, light intensity, pressure, chemical and biological contaminations, etc. For basic environmental sensing, the most important requirements for any type of sensor are reasonable measurement range, nominal response time, easy integration with read out circuits, and cost effectiveness^1^,^2^. Relative humidity is among the most important and common environmental factors that is widely studied in different areas of research. Measurement and control of relative humidity is crucial in sensitive environments like medicine, health, food, and industry^3^. Different types of sensor structures and transducing techniques like surface acoustic waves^4^,^5^, interdigitated transducers^6^, field effect transistors^7^, quartz crystal based transducer^8^, and other structures^9^,^10^,^11^,^12^ have been used for humidity sensing. A wide variety of materials have been used for the fabrication of humidity sensors including simple and complex polymers, metal oxides, carbon, graphene, and other composites^13^,^14^,^15^,^16^,^17^. Capacitive, resistive, and impedance based sensors are the best choice for general applications requiring nominal performance as they are easy to fabricate, characterize, and cost much lower than the complex sensors^1^,^2^.

In quest of good performance and low cost, researchers often tend to get inspiration from nature or use some basic off the shelf house hold materials for certain applications. This allows them to obtain properties and performance that are not usually possible even with expensive laboratory synthesized materials and chemicals. For example, carbonized chicken eggshell membranes with 3D architectures have been used for the fabrication of high-performance electrode materials for supercapacitors owing to the suitable properties of the membrane^18^. Single cell thick onion membrane has been used to make artificial muscles that can both contract and expand upon bending^19^. The physical properties of the onion membrane suggested that it can be used for the certain application. Invertase-nanogold clusters decorated onion membrane has been used for fluorescence-based sucrose sensor^20^. Mimosa leaflets were used as the mold for the fabrication of bio-inspired touch sensor with quite reasonable response^21^. The surface structure of the leave suggested them to be ideal for touch sensing. A new concept for selective chemical detection has been presented using bioinspired photonic nanostructures on the wing scales of Morpho butterflies possessing acute chemical-sensing capabilities^22^. Lead pencil pattern based strain and gas sensors have been reported recently showing stable and repeatable response^23^. The pencil lead contains carbon that is both conductive and porous plus it is bendable for a specific pencil type, making it a suitable candidate to be tested for the mentioned application. Similarly, a multi-sensory paper skin platform has been fabricated completely using general household items like tissue papers, conductive pen, copper tape, aluminum foil, Teflon tape, etc. Different sensors on the platform included temperature, humidity, PH, and force sensors^24^. The bio and non-biomaterials available around us in daily life have good potential to be employed in unique applications as they can open new possibilities towards bio-compatible and environmental friendly degradable electronic devices.

In addition, further high level research is underway on wearable, implantable and transferrable devices^25^. Electronic device fabrication on very thin substrates is of high interest in this regard as these substrates exhibit high conformability, bendability, and lightness; which are crucial attributes for biological tissues sensing and wearable or implantable devices^26^. Most common methods of transfer printing of devices include water soluble sacrificial layers^27^, PDMS stamping^28^,^29^, and gecko printing^30^. A solution-based *in-situ* transfer method has also been used for hybrid thin film fabrication and transfer onto arbitrary substrates^31^. A high-yield two-step transfer printing method for large-scale fabrication of organic single-crystal devices on arbitrary substrates has been reported^32^. Nanowire devices have also been fabricated on diverse substrates by simple transfer printing methods^33^,^34^.

Researchers are trying to combine the low cost, high performance, environmental friendliness, and transferrable printed electronic devices to achieve the best results. The conventional transfer printing methods are usually very complex involving multiple steps and are highly sensitive with high chances of device malfunction while transferring^35^. Moreover, the substrates yet used have only one function to support the device printed on them with no intrinsic sensing properties demanding additional functional layers to be printed on top. These polymeric thin film substrates are also not always bio-compatible.

In this research work, a single cell thick onion membrane has been employed as a humidity sensing active layer for the fabrication of an impedance based sensor while it also doubles as the device substrate owing to its high robustness and easy handling. Onion membranes are composed of plant cells which means they have an outer cell wall in addition to a cell membrane. This allows them to retain their structure even in case of deformation in the inner cell body upon dehydration^36^. The cell boundary acts as an osmotic membrane that is permeable to external solutions until an equilibrium is reached^36^. This phenomenon makes the membrane suitable to absorb permeable solvents including water and can be used as a humidity sensing layer while its ultra-low thickness allows it to be transferred to arbitrary shaped substrates with high conformability. The transfer process is single step, very simple, and quite safe for the device. The overall device, thus, is a biocompatible, biodegradable, and disposable organic device that can be transferred to any arbitrary surface with a pretty good performance characteristics of relative humidity sensing from 0% RH to 80% RH.

## Results

### Physical Characterizations

While using an ultrathin substrate for the device, a major concern is the possible damage to the fragile substrate during the different fabrication steps. Possible steps that could damage the membrane during this process were pressure applied when the electrode patterns were printed through screen printing, heating of the device to cure the epoxy contacts, and drawing the electrodes using the conductive pen by hand. Optical microscopy was performed after each step to make sure that the onion membrane was intact and the quality of printed electrodes is good. The microscopic images are presented in Supplementary Figure S1 which show that the membrane was intact at all the steps and continuous electrodes were printed successfully without any defects.

2D and 3D surface profiles of the sensors were recorded to check the physical properties of both sides of the membrane and to estimate the thickness of the membrane and the height of printed electrodes. The surface nano profile results are presented in Supplementary Figure S2. The surface profiles of the membrane indicate that the surface of the hydrophilic side has a higher average roughness of 4.29 μm compared to 2.78 μm for the hydrophobic side. Also, it is known that more is the surface roughness, larger is the active area to volume ratio, resulting in higher device sensitivity^15^. The 3D profiles with printed electrode was used to estimate the height of the electrode that was measured to be ~8 μm. The length of a single cell was 270 μm while the width was 70 μm.

**Figure 1:**
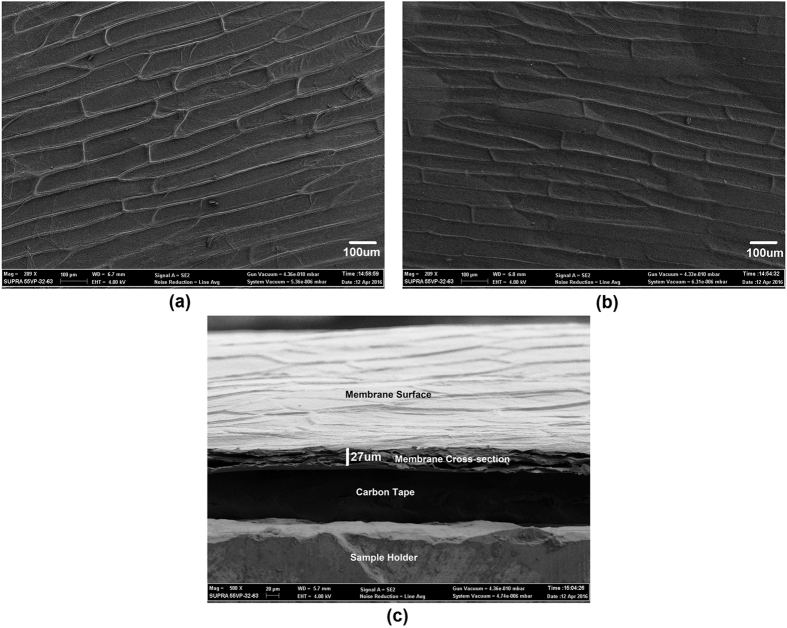
SEM images of the film showing surface morphology of the (**a**) Hydrophilic side of the membrane, (**b**) Hydrophobic side of the membrane, and (**c**) Cross-section of the membrane.

Surface and cross-sectional SEM images were taken to accurately observe the surface morphology of the two sides of the membrane and to calculate the thickness of the dried single cell thick membrane respectively. The surface SEM characterization results are presented in Fig. 1(a,b). The images indicate that the hydrophilic side of the membrane has higher visible surface roughness with cell boundaries bulging out of the plane whereas the surface of the hydrophobic side is comparatively smoother with very little bulging on the cell boundaries. This is due to the difference in the properties and structure of cell membrane present on both sides, thus, giving unique properties to each of them. The cross sectional SEM image presented in Fig. 1(c) shows the thickness of the dried layer. The results indicate that the thickness of the dried membrane is ~27 μm that is exactly according to the expectations.

### Sensor Performance Characterization

**Figure 2:**
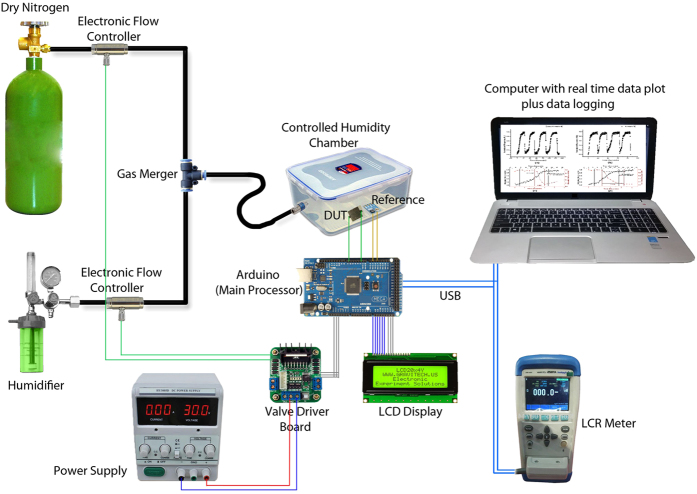
Detailed schematic design of the in-house developed characterization setup used for generation of a controlled humidity environment for output response measurement of sensors and automatic data logging and plotting in computer.

The sensors were characterized for their response towards changing humidity levels of a controlled environment by recording their impedance (R + 2πfL + 1/2πfC) variation and the trend it follows. The setup was designed and fabricated in-house to accurately and reliably record the data^37^. The schematic diagram of the characterization setup is presented in Fig. 2. The level of percentage relative humidity inside the sealed chamber was controlled by using an automatic feedback controller. Dry Nitrogen (N_2_) was used for decreasing the relative humidity while atomized water from an office desktop humidifier was used to increase the level of humidity inside the chamber. The flow rate of both the gases was controlled by electronic valves whose position was determined by the output of the feedback flow controller. The real time readings of the humidity level inside the chamber were displayed on an LCD and were also sent to computer through USB communication for automatic data logging.

**Figure 3:**
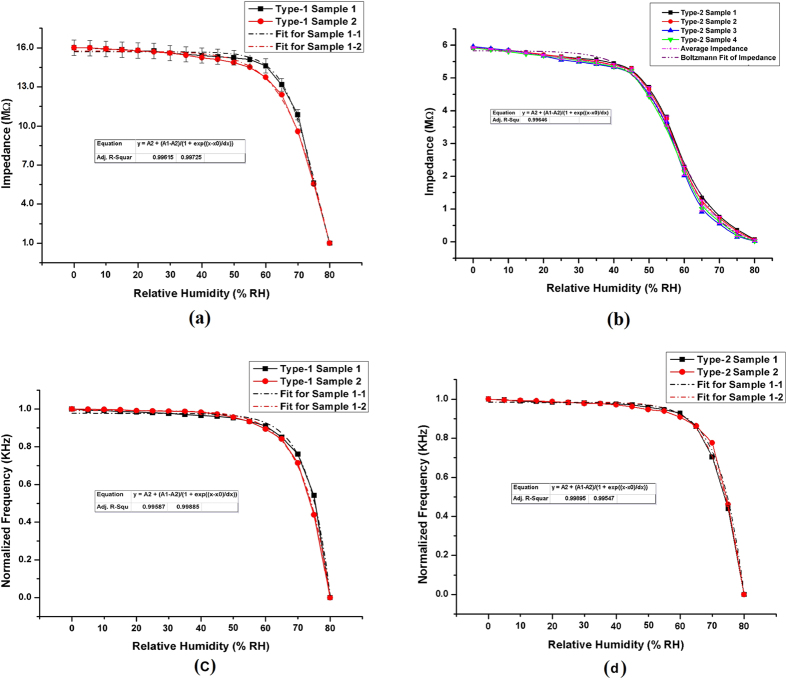
Humidity sensor response (**a**) impedance response of type-1 sensors and (**b**) impedance response of type-2 sensors, (**c**) normalized frequency response of type-1 sensors, and (**d**) normalized frequency response of type-2 sensors.

The humidity level inside the chamber was changed by ~5 ± 1% RH per step through a manual user input and was maintained at that point until the impedance readings of the sensors were stable. The time to stabilize impedance readings of the sensors was given to compensate for the difference in response times of the reference sensor and the fabricated sensors and to overcome the hysteresis effect of the sensors. The sensors’ response was taken for the relative humidity range of 0% RH to 80% RH where the temperature for the whole experiment was maintained at ~20 °C. The impedance response of the sensors is presented in Fig. 3(a,b).

**Figure 4:**
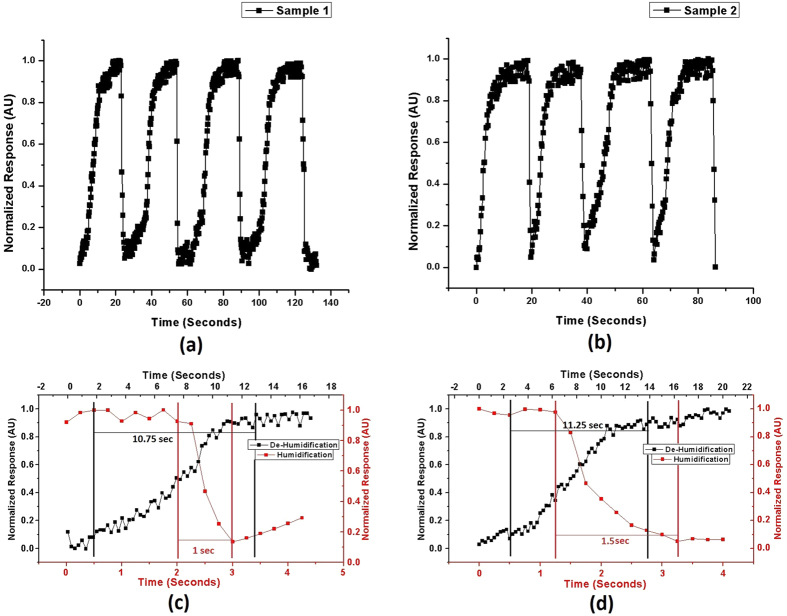
Response time curves of (**a**) Type-1 sensor, (**b**) Type-2 sensor, (**c**) Zoomed-in single cycle for type-1 sensor, and (**d**) Zoomed-in single cycle for type-2 sensor.

A square wave oscillator circuit was then designed based on a *555 timer* in an astable mode^38^ to convert the response of sensors to change in frequency that could be easily read by simple electronic readout circuits. This enables the sensors to be used in real life applications^39^. The circuit for frequency conversion and the actual image of the other electronic circuitry is presented in Supplementary Figure S3. The output frequency response of the sensors is presented in Fig. 3(c,d). Response time measurements of the sensors were taken by choosing two different flow rate combinations within the measurement range of humidity and having a reasonable difference in values. The readings were taken by switching the flow rates repeatedly after the impedance readings got stable. The measurement resolution of the setup was 0.1 s. The response time curves for the sensors are presented in Fig. 4.

## Discussion

Onion membranes are very good insulators when they are completely dried and have a very high impedance value. The impedance of the membrane depends largely upon water content inside the cells. The water adsorbed by the membrane can dissolve the dried organic materials inside the cells and provide a path for the flow of current. The water itself is also a comparatively very good conductor and its relative permittivity value also differs significantly from that of the dried membrane. Water is adsorbed or released by the cells following diffusion and osmosis principles through the cell membranes. Whenever there is a difference in the water contents level inside the cells and the outside environment, the membrane tries to gain equilibrium through diffusion and osmosis. The membrane keeps on releasing to or adsorbing the water vapors from the outer environment until an equilibrium state is reached between the inner cell environment and the outer environment. This fact is taken in to consideration and it serves as the basic working principle for the fabricated humidity sensors.

The impedance response results presented in Fig. 3(a,b) indicate that the overall impedance of both types of the sensors decrease non-linearly with increasing relative humidity level. For type-1 sensors, two different samples were fabricated and characterized for 3 sets of readings each. For type-2, four identical sensors were fabricated using screen printing. Figure 3(a) shows that in case of type-1 sensors, the overall change in impedance is 16 MΩ to 2 MΩ for 0% RH to 80% RH. As the sensors were drawn by hand, they were not identical and show higher uncertainty and lower precision and repeatability. Figure 3(b) shows that in case of type-2 sensors, the impedance changes from ~6 MΩ to ~20 KΩ for 0% RH to 80% RH. Multiple readings for different samples of type-2 sensors are almost similar with much higher precision and repeatability as the sensors were exactly identical as presented in supplementary Figure S2. The impedance response curves were fitted using Boltzmann second order curve fitting equations with an R^2^ value greater than 0.99 in all cases. This shows that the response of the sensors can be easily converted to relative humidity with quite high accuracy by solving the equation.

The onion membrane has a natural tendency to absorb water when exposed to high humidity environments after complete de-hydration. The maximum quantity of water that can be absorbed depends upon when the equilibrium state is reached between the internal and external environments of the cell. The rehydration property and the robustness of membrane was tested by hot air blow drying of the membrane and then rehydrating it through direct dipping in water. The process was repeated for 10 times each over the course of 15 days and the membrane remained unharmed, regaining its original soft state upon exposure to water. This proves the robustness of the membrane that makes it a suitable candidate to be used as the substrate as well in addition to the active layer of the sensor. The onion cells may have died after being dry for a long time but their water absorption capability remains intact.

The sensors were also tested to analyze their output in terms of frequency, rather than impedance, by using an oscillator circuit. The output responses in terms of normalized frequency vs relative humidity are presented in Fig. 3(c,d). Same procedure of impedance measurement was used to record frequency based readings for the sensors. The frequency output of the circuit also decreases by increasing the humidity level of the chamber similar to the case of impedance. The response curves were fitted using exponential decay curve fitting equations that are displayed on the graphs showing the average R^2^ values of greater than 0.99 in all cases. The results indicate that the sensors’ output can be reliably converted in to a recognizable form that can be easily accepted as an input by a readout circuit to make them suitable for real life humidity sensing applications.

The response time (10–90% of the maximum value) and recovery time (90–10% of the maximum value) of the sensors were recorded by choosing two different humidity levels within the measurement range having a reasonable difference in values by adjusting the flow rates of the gases. The response time graphs for both types of sensors with a resolution of 0.25 s are presented in Fig. 4. The curves indicate excellent capability of the sensors to respond towards increasing and decreasing humidity levels with reasonable response and recovery times. The response time estimated in case of type-1 sensor was 1 second while for type-2 sensor, it was 1.5 seconds. The recovery time for type-1 sensor was approximately 10.75 seconds and for type-2, it was 11.25 seconds. The results indicate that there is negligible difference in the values for both types showing that the hydrophobic side plays the major role in both water adsorption and desorption. Both the sensors showed excellent performance statistics in terms of sensitivity and response time.

**Figure 5:**
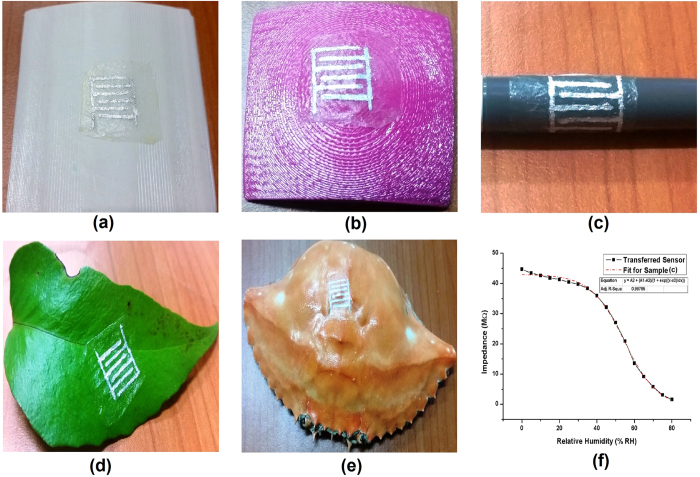
Images of the sensor fabricated on standalone membrane on various arbitrary shaped substrates (**a**) Curved substrate with diameter 16 cm, (**b**) Curved substrate with diameter 12 cm, (**c**) Ballpoint with diameter 8 mm (**d**) Plant leaf, (**e**) Crab shell, and (**f**) Impedance response of sensor transferred to substrate **b**.

As the last step of this research work, a sensor was fabricated on a standalone onion membrane by drawing the IDT’s on the hydrophilic side of the membrane as presented in Supplementary Fig. S1(g–1). The fabricated sensor was then transferred/mounted onto various arbitrary shaped surfaces as target substrates. The substrates included flat surfaces, curved surfaces with different curvature radii, and arbitrary uneven surfaces. For transferring the device, the target surface was first wetted by water droplet and the non-printed side of the membrane was carefully placed on the wet surface. The membrane readily absorbs the water on the target surface upon coming in contact and becomes soft and highly flexible. After the membrane was homogenously and conformably transferred to the target surface, the sensor was blow dried using hot air. This removes any water adsorbed by the membrane and makes it ready for humidity sensing. The images of the sensor attached to various substrates are presented in Fig. 5(a–e). The output response curve of the sensor after transferring to the substrate with 12 cm curvature diameter is presented in Fig. 5(f). The surface with minimum curvature radius/maximum surface curvature was chosen to record readings of the device as it proves that the device will also work on the surfaces with flatter surfaces. The figure clearly shows that the device can be transferred to any arbitrary shaped surface with high conformity and without damaging the sensor. The overall intrinsic impedance of the sensor comparatively increased after bending but the humidity sensing behavior was not effected showing similar response curves. Possible reason for increase in intrinsic impedance is formation of micro-cracks in the electrodes after bending.

As a conclusion of this study, we can state that the response of transferrable biocompatible sensors fabricated by using a single cell thick onion membrane as both the substrate and the active layer for humidity sensing devices exhibit remarkable performance. Dried onion membrane is a pure insulator with very high impedance but it can absorb water from the environment through diffusion and osmosis process. As the water content inside the cells increases, the impedance of the membrane decreases, and this phenomenon is used as the key to use the membrane as a humidity sensing layer. The AC impedance of the sensors was measured between 0% RH and 80% RH at 10 kHz test frequency with maximum change in impedance from 16 MΩ to 2 MΩ for type-1 and 6 MΩ to 20 KΩ for type-2. The fabricated sensors showed good response and recovery times of ~1.5 seconds and ~11 seconds respectively. Their output response in terms of frequency change using an oscillator conversion circuit is also appreciable. The single cell thick membrane was tested for 10 drying and rehydration cycles per day for 15 days and the membrane remained intact and undamaged after all the tests were completed. The fabricated sensors show high reliability and robustness with excellent repeatability and life time. The device transfer results indicate that the ~30 μm thin membrane is ideal to be used as the substrate replacing PDMS and other ultra-thin substrates for the electronic devices that need to be conformably transferred to an arbitrary target surface. Possible application areas of this research include a wide variety of research works. The ultra-thin membrane can be used for electronic skin applications as the device can be conformably transferred to any substrate. It also has possible applications in implantable sensors as the membrane is a bio-material and can be safely implanted for inner body parameters monitoring. The membrane can also serve as an environmental friendly disposable substrate for environmental monitoring applications. Further detailed studies can be performed and different properties of the membrane can be explored by chemically or physically treating the membrane for example use of sulfuric acid treatment for removing hemicellulose^19^ to achieve desired properties. It opens a new door for printed electronic researches where the onion membrane can be employed as a substrate, as an active layer, as an insulating barrier layer when completely dried, and so on.

## Methods

### Materials

Fresh edible onion was taken and a single cell thick membrane was removed by carefully peeling it off with sharp nose tweezers. The IDT electrode patterns were fabricated by screen printer using bio-compatible water soluble Ag nanoparticles ink purchased from PARU with particles diameter range of 20~200 nm, Ag contents of 80~88 wt% and typical resistance of 2.0 mΩ/□/mil. Electroninks Circuit Scribe Conductive ink pen with ~2 Ω/cm conductivity was used for the hand drawn IDT patterns on the membrane. Circuitworks conductive epoxy CW2400 was used for connecting wires. PLA was used for FDM based 3D printing of the plastic support frame for membrane. Dry Nitrogen (N_2_) gas was used as de-humidifier in the characterization. De-ionized (DI) water was used for cleaning purposes.

### Equipment

The fabricated sensors and the onion membrane were observed under optical microscope using Olympus BX51 M computerized HR colored microscope. The 2D and 3D profiles of the film and sensors were observed using *NanoView NV2200* high accuracy 3D nano optical non-contact surface profiler. Contact angle measurements were done using *Phoenix Phx-300 touch* contact angle analyzer system. Scanning electron microscopy (SEM) was performed for surface and cross-sectional images of the membrane using *Carl Zeiss Supra 55VP* high end microscope. *HTU21D* with a resolution of 0.04% RH and an accuracy of ±2% RH was used as the reference humidity sensor for feedback to the digital controller and also to determine the current level of humidity inside the chamber. The temperature coefficient for the reference sensor is −0.15% RH/°C and the response time is <5 s. Arduino *MEGA 2560* was selected as the main processing board for interfacing with sensors and computer. The AC impedance of the all the sensors was measured at 10 kHz test frequency input with 0.6 V_rms_ magnitude using the *Applent AT825 digital LCR Meter.*

### Sensor Fabrication Process

**Figure 6:**
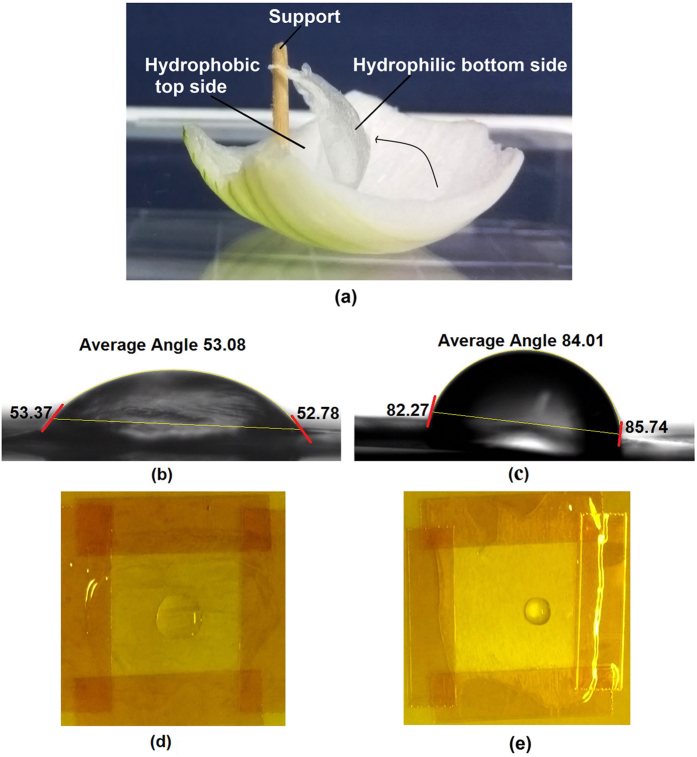
Hydrophilic and hydrophobic sides of the membrane with (**a**) showing the peeled off membrane mentioning the location of the two sides, (**b**) showing the water droplet contact angle at the hydrophilic side, (**c**) contact angle at the hydrophobic side, (**d**) image of droplet on hydrophilic side, and (**e**) image of droplet on hydrophobic side (without plasma treatment).

Fresh onion was cut and the thick top dead layer was removed. The single cell thick membrane was then peeled off carefully using sharp tweezers and was rinsed and washed in DI water to remove any impurities. For type-1 sensors, with both sides exposed to the environment, the cleaned membrane was mounted in to a 3D printed circular frame of plastic. The frame was tightened to remove any wrinkles from the surface of the free-standing membrane. The top side of the membrane is highly hydrophobic while the bottom side is comparatively hydrophilic as presented in Fig. 6(a). The hydrophilic side of the membrane was identified by contact angle measurement of water droplet on both surfaces. The results of contact angle measurement are presented in Fig. 6(b,c) showing the hydrophilic bottom side and the hydrophobic top side respectively. It can be seen that the average contact angle of the water droplet on the hydrophilic side (53°) is ~30° lower than that of the hydrophobic side (84°). The optical images, as presented in Fig. 6(d,e), for the same sized water droplet (15 μl) also show that the droplet spreads on the hydrophilic side while there is no spreading in case of the hydrophobic side.

**Figure 7:**
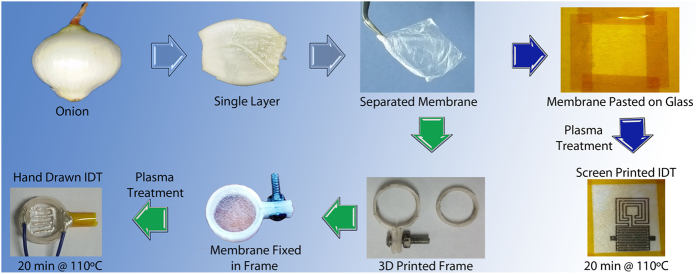
Step by step process flow diagram with green arrows showing the fabrication of type-1 sensors and blue arrows showing the fabrication of type-2 sensors.

To further improve the hydrophilic property of the membrane to assist the printing of electrodes and also for substrate cleaning, ozone plasma treatment was carried out for 3 minutes. Plasma treatment helps remove the organic contaminants on the surface of the substrate causing a reduction in contact angle^40^,^41^. After the treatment, interdigitated transducer electrodes (IDT) were drawn by hand on the hydrophilic side of the membrane using a soft tip conductive pen. It was made sure that all the process was completed with-in 20 minutes so that the membrane does not get dry and brittle. After drawing the IDT patterns and connecting the connecting wires, the sensors were dried at 110 °C for 30 minutes to cure the epoxy contacts. This step also removes all the water trapped inside the membrane cells. The type-1 sensors were ready to be tested at this point. The type-2 sensors were fabricated by pasting the cleaned onion membrane on to a glass slide with the hydrophilic side facing upwards. Wrinkles were removed using a damp cotton bud. The membrane was fixed at its position using Teflon masking tape. The sample was plasma treated and then the silver IDT patterns were printed onto the membrane using screen printer^42^. Connecting wires were attached using conductive epoxy and the samples were dried at 120 °C for 20 minutes. For free standing membranes as in case of type-1 sensors, the membrane was cracked at 120 °C due to excessive cell shrinking at a higher temperature as they were already mounted in a high tension wet state. Therefore, a lower temperature and longer time were selected for type-1 drying. The step by step fabrication process flow diagram is presented in Fig. 7.

## Additional Information

**How to cite this article**: Sajid, M. *et al*. Bio-compatible organic humidity sensor transferred to arbitrary surfaces fabricated using single-cell-thick onion membrane as both the substrate and sensing layer. *Sci. Rep.* **6**, 30065; doi: 10.1038/srep30065 (2016).

## Supplementary Material

Supplementary Information
