# Supplementary material for: Bio-compatible organic humidity sensor transferred to arbitrary surfaces fabricated using single-cell-thick onion membrane as both the substrate and sensing layer
Source: Sci Rep. 2016 Jul 20;6:30065. doi: 10.1038/srep30065 (PMC4951809; doi:10.1038/srep30065)
Supplement: Supplementary Information [file srep30065-s1.pdf]

Supplementary Information for

Bio-compatible organic humidity sensor transferred to arbitrary surfaces fabricated using single-cell-thick onion membrane as both the substrate and sensing layer

Memoon Sajid[1], Shahid Aziz[1], Soo Wan Kim[1], Kim Go Bum[1], Jeongdai Jo[2], Kyung Hyun Choi[1,]*

[1]Department of Mechatronics Engineering, Jeju National University, Jeju 690-756, South Korea

[2]Korean Institute of Machinery and Materials, Yuseong-Gu, Daejeon 305-343, Republic of Korea

[*]E-mail: amm@jejunu.ac.kr

[*]Phone: +82-64-754-3713

[*]Fax: +82-64-752-3174

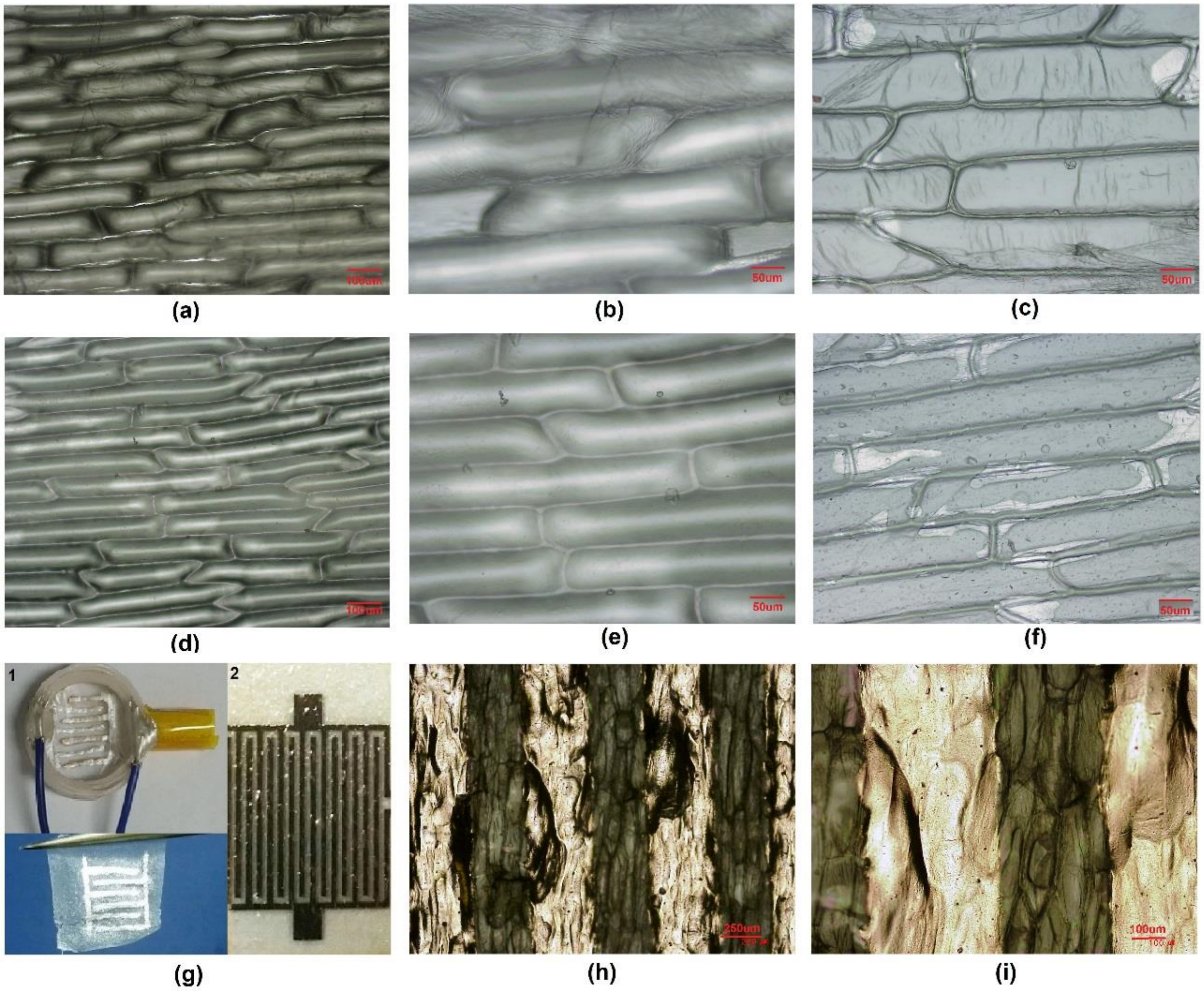

Figure S1 | Microscopic images of hydrophilic side of membrane at **(a)** 10x magnification before drying, **(b)** at 20x before drying, **(c)** at 20x after drying, **(d)** hydrophobic side at 10x before drying, **(e)** at 20x before drying, **(f)** at 20x after drying, **(g)** Images of fabricated sensors showing 1: Type-1 and 2: Type-2 sensors, **(h)** Membrane with printed electrodes at 5x, and **(i)** at 10x magnification.

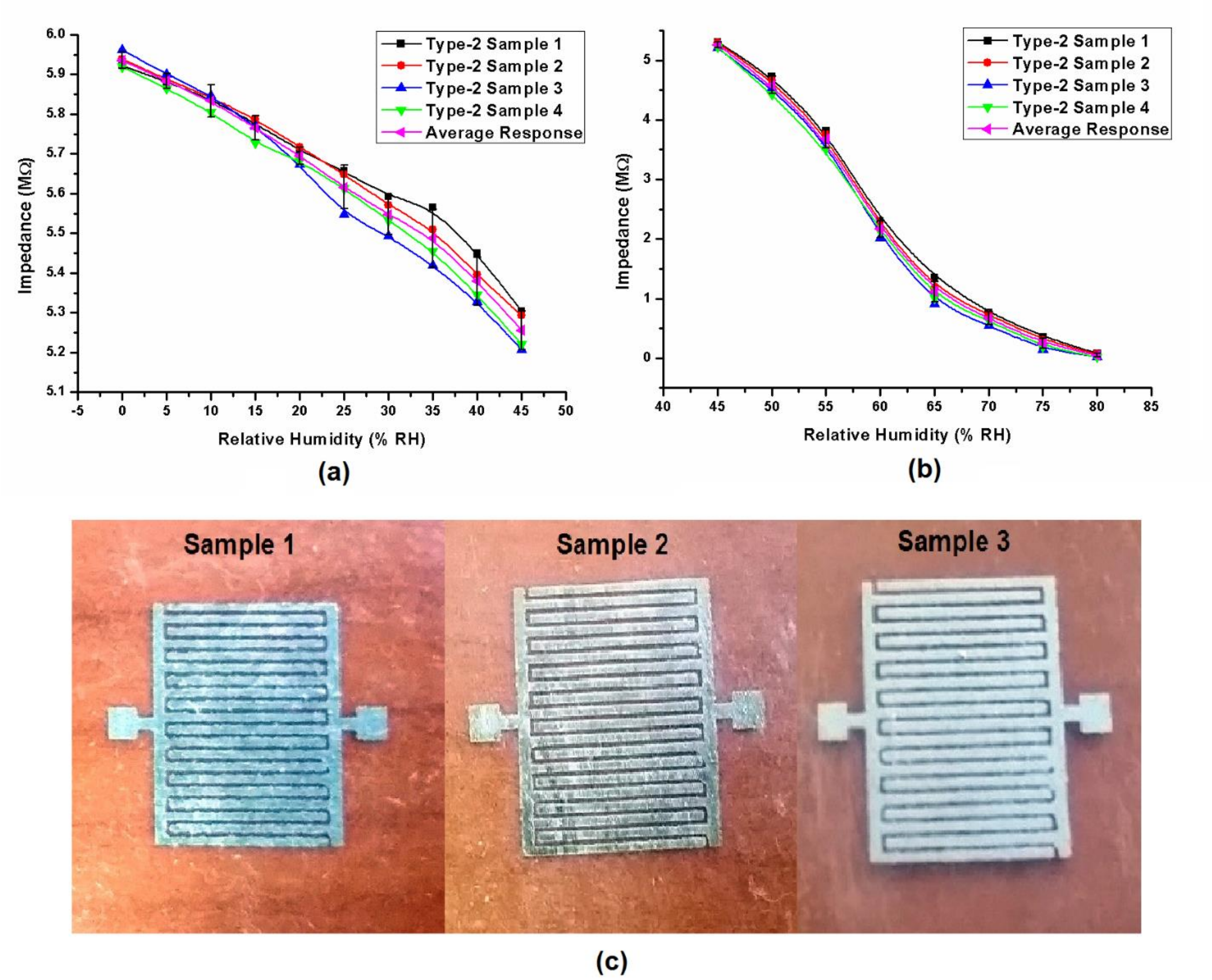

Figure S2 | Impedance response of type-2 sensors (a) zoomed in for 0% RH to 45% RH, **(b)** 45% RH to 80% RH showing good repeatability and low uncertainty, and **(c)** Images of multiple samples of type-2 sensors fabricated using screen printing of electrodes on to the onion membrane attached to a glass substrate.

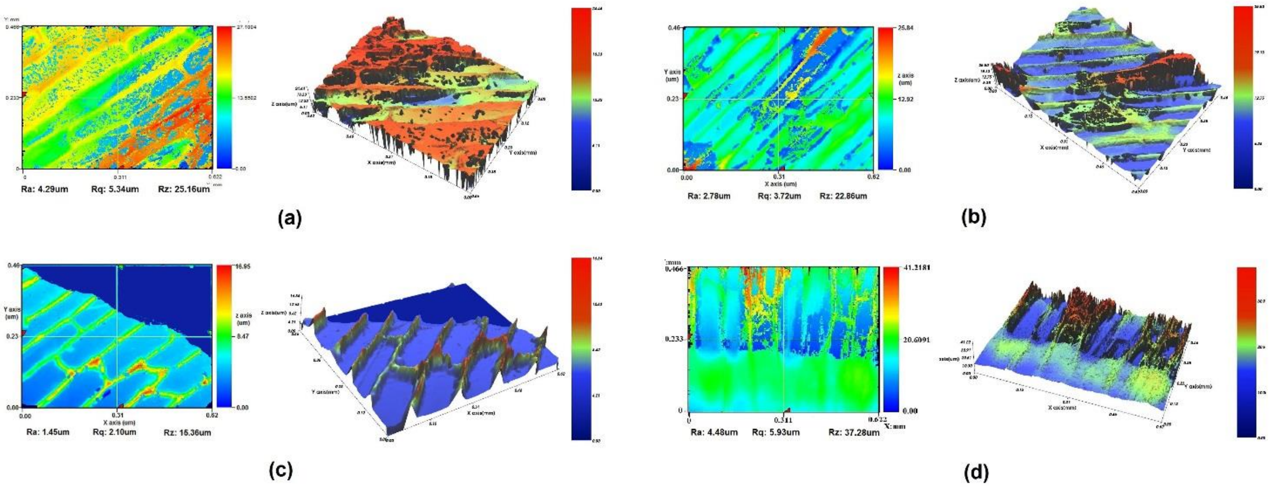

Figure S3 | 2D and 3D profiles of the membrane with the roughness parameters of (a) Hydrophilic side, **(b)** Hydrophobic side, **(c)** Membrane mounted on glass with boundary scan measurement for height calculation, and **(d)** Membrane with printed electrode.

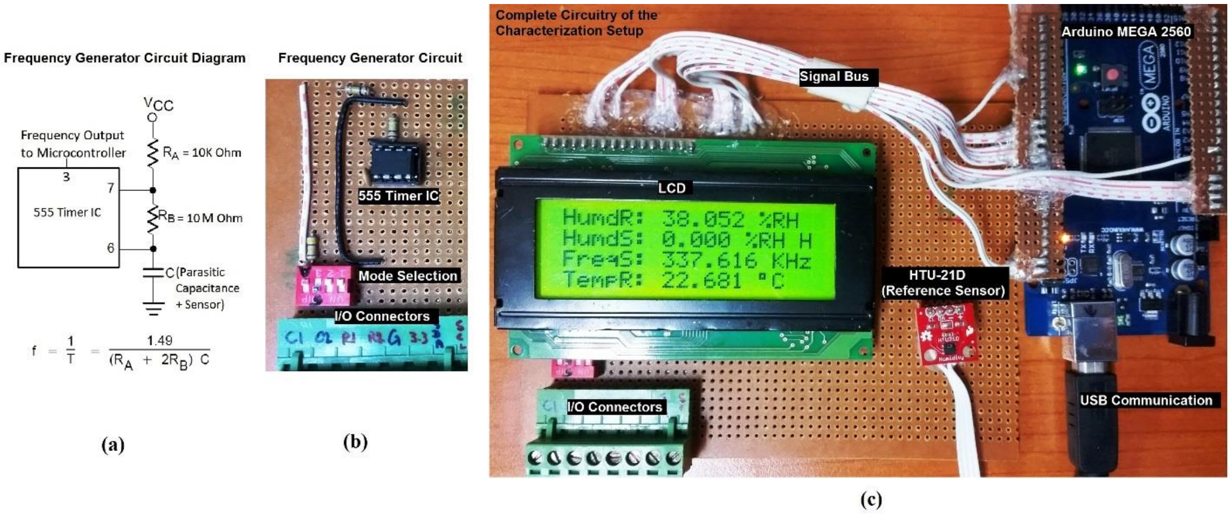

Figure S4 | Sensor output to frequency conversion circuit. (a) Circuit diagram and frequency conversion formula, **(b)** Image of the implemented circuit, and **(c)** The image of all the electronic circuits and interfacing.

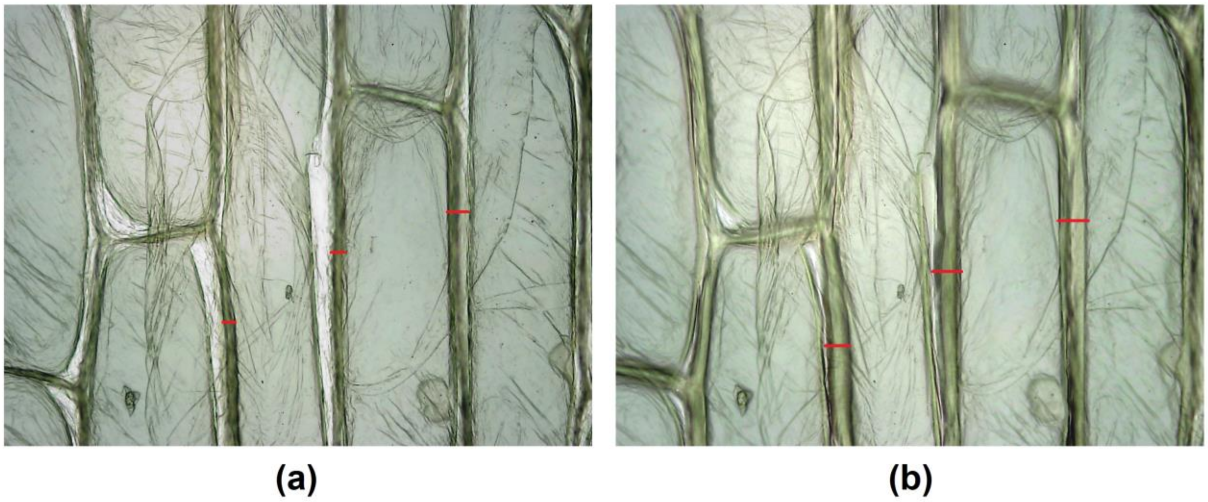

Figure S5 | Onion membrane images at 20x showing (a) the membrane exposed to 0% RH environment and **(b)** the membrane exposed to 50% RH environment.

It can be clearly noted that the dry membrane has gone through shrinkage in cell membranes while the one with absorbed moisture has swelled cell membranes indicating the presence of water content inside.
